# A gene based approach to test genetic association based on an optimally weighted combination of multiple traits

**DOI:** 10.1371/journal.pone.0220914

**Published:** 2019-08-09

**Authors:** Jianjun Zhang, Qiuying Sha, Guanfu Liu, Xuexia Wang

**Affiliations:** 1 Department of Mathematics, University of North Texas, Denton, TX, United States of America; 2 Department of Mathematical Sciences, Michigan Technological University, Houghton, MI, United States of America; 3 School of Statistics and Information, Shanghai University of International Business and Economics, Shanghai, China; Brigham and Women’s Hospital and Harvard Medical School, UNITED STATES

## Abstract

There is increasing evidence showing that pleiotropy is a widespread phenomenon in complex diseases for which multiple correlated traits are often measured. Joint analysis of multiple traits could increase statistical power by aggregating multiple weak effects. Existing methods for multiple trait association tests usually study each of the multiple traits separately and then combine the univariate test statistics or combine p-values of the univariate tests for identifying disease associated genetic variants. However, ignoring correlation between phenotypes may cause power loss. Additionally, the genetic variants in one gene (including common and rare variants) are often viewed as a whole that affects the underlying disease since the basic functional unit of inheritance is a gene rather than a genetic variant. Thus, results from gene level association tests can be more readily integrated with downstream functional and pathogenic investigation, whereas many existing methods for multiple trait association tests only focus on testing a single common variant rather than a gene. In this article, we propose a statistical method by Testing an Optimally Weighted Combination of Multiple traits (TOW-CM) to test the association between multiple traits and multiple variants in a genomic region (a gene or pathway). We investigate the performance of the proposed method through extensive simulation studies. Our simulation studies show that the proposed method has correct type I error rates and is either the most powerful test or comparable with the most powerful tests. Additionally, we illustrate the usefulness of TOW-CM based on a COPDGene study.

## Introduction

Complex diseases are often characterized by many correlated phenotypes which can better reflect their underlying mechanism. For example, hypertension can be characterized by systolic and diastolic blood pressure [1]; metabolic syndrome is evaluated by four component traits: high-density lipoprotein (HDL) cholesterol, plasma glucose and Type 2 diabetes, abdominal obesity, and diastolic blood pressure [2]; and a person’s cognitive ability is usually measured by tests in domains including memory, intelligence, language, executive function, and visual-spatial function [3]. Also, more and more large cohort studies have collected or are collecting a broad array of correlated phenotypes to reveal the genetic components of many complex human diseases. Therefore, by jointly analyzing these correlated traits, we can not only gain more power by aggregating multiple weak effects, but also understand the genetic architecture of the disease of interest [4].

Even though genome-wide association studies (GWASs) have been remarkably successful in identifying genetic variants associated with complex traits and diseases, the majority of the identified genetic variants only explain a small fraction of total heritability [5]. Furthuer, a gene is the basic functional unit of inheritance whereas the GWAS are primarily focused on the paradigm of single common variant. However, most published GWASs only analyzed each individual phenotype separately, although results on related phenotypes may be reported together. Large-scale GWAS of complex traits have consistently demonstrated that, with few exceptions, common variants have moderate-to-small effects. Therefore, it is important to identify appropriate methods that fully utilize information in multivariate phenotypes to detect novel genes in genetic association studies.

In GWAS, several methods have been developed for multivariate phenotypes association analysis [3] to test association between multivariate continuous phenotypes and a single common variant. To our knowledge, current multivariate phenotypes association methods can be roughly classified into two categories: univariate analysis and multivariate analysis. Univariate analysis methods perform an association test for each trait individually and then combine the univariate test statistics or combine the p-values of the univariate tests [6–9]. Even though such methods are computationally efficient, they neglect the omnipresent correlation between individual phenotypes and may reduce the power compared to multivariate analysis. Multivariate analysis methods jointly analyze more than one phenotype in a unified framework and test for the association between multiple phenotypes and genetic variants. Multivariate analysis methods include multivariate analysis of variance (MANOVA) [10], linear mixed effect models (LMM) [11], and generalized estimating equations (GEE) [12]. Another special approach is to consider reducing the dimension of the multivariate phenotypes by using dimension reduction techniques. The common method for dimensionality reduction is principal component analysis (PCA) [13] which essentially finds the combination of these phenotypes and assumes that the transformed phenotypes are independent. The limitation of this method is that it can not properly account for the variation of phenotypes or genotypes. It is also hard to interpret the meaning of principle components of the multivariate phenotypes, especially in practice.

Recent studies show that complex diseases are caused by both common and rare variants [14–20]. Gene-based analysis requires statistical methods that are fundamentally different from association statistics used for testing common variants. It is essential to develop a novel statistical method to test the association between multiple traits and multiple variants (common and/or rare variants). In this article, we develop a statistical method to test the association between multiple traits and genetic variants (rare and/or common) in a genomic region by Testing the association between an Optimally Weighted combination of Multiple traits (TOW-CM) and the genomic region. TOW-CM is based on the score test under a linear model, in which the weighted combination of phenotypes is obtained by maximizing the score test statistic over weights. The weights at which the score test statistic reaches its maximum are called the optimal weights. We also use extensive simulation studies to compare the performance of TOW-CM with MANOVA [10], multi-trait sequence kernel association test MSKAT [21] and minimum p-value [22]. Simulation studies demonstrate that, in all the simulation scenarios, TOW-CM is either the most powerful test or comparable to the most powerful test among the four tests. We also illustrate the usefulness of TOW-CM by analyzing a real COPDGene study.

## Methods

We consider a sample with *n* unrelated individuals. Each individual has *K* (potentially correlated) traits and has been genotyped at *M* variants in a considered region (a gene or a pathway). Denote *y*_*ik*_ as the *k*^*th*^ trait value of the *i*^*th*^ individual and *x*_*im*_ as the genotype score in additive coding of the *i*^*th*^ individual at the *m*^*th*^ variant. Let ***Y*** = (*Y*_1_, ⋯, *Y*_*K*_) denote the random vector of *K* traits and ***X*** = (*X*_1_, ⋯, *X*_*M*_) denote the random variable of the genotype score at *M* variants for these *n* individuals where *Y*_*k*_ = (*y*_1*k*_, ⋯, *y*_*nk*_)^*T*^ and *X*_*m*_ = (*x*_1*m*_, ⋯, *x*_*nm*_)^*T*^. Consider a linear combination of ***Y*** denoted as $Yw=\sum_{k=1}^{K}w_{k}Y_{k}$, where *w* = (*w*_1_, ⋯, *w*_*K*_)^*T*^.

We model the relationship between the combination of multiple continuous traits with the *M* genetic variants in the considered region using the linear model

$$\begin{array}{c} \sum_{k=1}^{M}w_{k}y_{ik}=\beta_{0}+\beta_{1}x_{i1}+\cdots +\beta_{M}x_{iM}+\epsilon_{i} \end{array}$$ (1)

where *β*_0_ is the intercept and ***β*** = (*β*_1_, ⋯, *β*_*M*_)^*T*^ is the corresponding vector of coefficients. To test the association between the combination of the multiple traits and the *M* genetic variants is equivalent to test the null hypothesis *H*_0_: ***β*** = 0 under Eq (1). We use the score test statistic to test *H*_0_: ***β*** = 0 under Eq (1). Let $P=I_{n}-\frac{1}{n}1_{n}1_{n}^{T}$ and then the test statistic is:

$$\begin{array}{c} S=U^{T}V^{-1}U \end{array}$$ (2)

where *U* = (*P****X***)′ *P**Y**w* and $V=\frac{1}{n}{\left(PYw\right)}^{{}^{\prime }}PYw{\left(PX\right)}^{{}^{\prime }}PX$. The score test can be rewritten as a function of *w*:

$$\begin{array}{c} S\left(w\right)=n*\frac{w^{{}^{\prime }}Y^{{}^{\prime }}PX{\left(X^{{}^{\prime }}PX\right)}^{-1}X^{{}^{\prime }}PYw}{w^{{}^{\prime }}Y^{{}^{\prime }}PYw} \end{array}$$ (3)

where *P* = *P*′ and *PP*′ = *P*. We propose to maximize *S*(*w*) to get the optimal weight and then define the statistic to evaluate the association between the optimally weighted combination of the target traits and test genetic variants.

When *D* = ***Y***′ *P**Y*** is positive definite, maximizing *S*(***w***) is equivalent to maximizing

$$\begin{array}{c} S\left(w\right)=\frac{w^{{}^{\prime }}LL^{-1}Y^{{}^{\prime }}PX{\left(X^{{}^{\prime }}PX\right)}^{-1}X^{{}^{\prime }}PYL^{-T}L^{T}w}{w^{{}^{\prime }}LL^{{}^{\prime }}w} \end{array}$$ (4)

where *L* is the lower triangular matrix obtained from the Cholesky decomposition of *D* = *LL*^*T*^. However, the matrix of *D* is usually not full rank because of existing correlation between multiple traits. If the matrix *D* is semi-positive define matrix, we introduce a ridge parameter λ_0_, for which we suggest the choice $\lambda_{0}=\sqrt{1/n}$, where *n* is the number of individuals in the testing data, and modify the adjustment to mitigate the effect of the non-positive matrix *D* in order to avoid the instability: *D* = ***Y***′ *P**Y*** + λ_0_*I*. Let *C* = *L*^−1^ ***Y***′ *P**X***(***X***′ *P**X***)^−1^ ***X***′ *P**Y**L*^−*T*^ and ***c*** be the eigenvector corresponding to the largest eigenvalue of the matrix *C*, then *S*(***w***) is maximized when *L*′(***w***) equals ***c***. Hence Eq (4) is maximized when *w^o^* = *L*^−*T*^ ***c***. In a special case, if all the traits we consider are independent and *M* = 1, we can get an analytical weight referred to [22]:

$$\begin{array}{c} w_{k}=\frac{{\left(PX\right)}^{T}\left(PY_{k}\right)}{{\left(PY_{k}\right)}^{T}\left(PY_{k}\right)}=\frac{X^{T}PY_{k}}{Y_{k}^{T}PY_{k}} \end{array}$$ (5)

for the *k*^*th*^ phenotype, *k* = 1, 2, 3, …, *K*. The Eq (5) is equivalent to $w_{k}=\frac{Corr(Y_{k},X)}{\sqrt{Y_{k}^{T}PY_{k}}}$ where the numerator is the correlation coefficient between the *k*^*th*^ phenotype *Y*_*k*_ and the genotypic variant *X* and the denominator can be viewed as the variance of the *k*^*th*^ phenotype *Y*_*k*_. It means that *w*_*k*_ has same direction with the correlation between the phenotype *Y*_*k*_ and the genotypic variant *X*, and puts big weight to the *k*^*th*^ trait when it has strong association with the genotypic variant and/or it has low variance.

We define the statistic to test an optimally weighted combination of multiple traits (TOW-CM), $Yw^{o}=\sum_{k=1}^{K}w_{k}^{o}Y_{k}$, as

$$\begin{array}{c} T=\frac{w^{o^{\prime }}Y^{{}^{\prime }}PX{\left(X^{{}^{\prime }}PX\right)}^{-1}X^{{}^{\prime }}PYw^{o}}{w^{o^{\prime }}Y^{{}^{\prime }}PYw^{o}} \end{array}$$ (6)

We use permutation methods to evaluate P-values of *T*. The TOW-CM method can also be extended to incorporate covariates. Suppose that there are *p* covariates. Let *z*_*il*_ denote *l*^*th*^ covariate of the *i*^*th*^ individual. We adjust both trait value *y*_*ik*_ and genotypic score *x*_*im*_ for the covariates by applying linear regressions. That is,

$$\begin{array}{cc} y_{ik} & =\alpha_{0}+\alpha_{1}z_{i1}+\cdots +\alpha_{p}z_{ip}+\epsilon_{ik}\;\;\;\text{and}\\ x_{im} & =\alpha_{0m}+\alpha_{1m}z_{i1}+\cdots +\alpha_{pm}z_{ip}+\tau_{im} \end{array}$$

Let ${\tilde{y}}_{ik}$ and ${\tilde{x}}_{im}$ denote the residuals of *y*_*ik*_ and *x*_*im*_, respectively. We incorporate the covariate effects in TOW-CM by replacing *y*_*ik*_ and *x*_*im*_ in Eq (6) by ${\tilde{y}}_{ik}$ and ${\tilde{x}}_{im}$. With covariates, the statistic of TOW-CM is defined as:

$$\begin{array}{c} T_{TOW-CM}{=T|}_{y_{ik}={\tilde{y}}_{ik},x_{im}={\tilde{x}}_{im}} \end{array}$$

## Comparison of tests

We compared the performance of our method (TOW-CM) with the following methods: 1) Multivariate Analysis of Variance (MANOVA) [10]; 2) Multi-trait Sequence Kernel Association Test (MSKAT) [21]; 3) Minimum p-value based on the p-values of the individual trait TOW [22] (denoted as minP).

## Simulation

In simulation studies, we use the empirical Mini-Exome genotype data including genotypes of 697 unrelated individuals on 3205 genes obtained from Genetic Analysis Workshop 17 (GAW17). Two differen type of variants (Common variants: minor allele frequency (MAF)>0.05 and Rare variants: MAF<0.05) are chosen from a super gene (Sgene) including four genes: ELAVL4 (gene1), MSH4 (gene2), PDE4B (gene3), and ADAMTS4 (gene4). The pattern of the allele frequency distribution of the Sgene is similar as the 3205 genes’ [22]. In our simulation studies, we generate genotypes based on the genotypes of 697 individuals in these four genes. The genotypes are extracted from the sequence alignment files provided by the 1,000 Genomes Project for their pilot3 study (http://www.1000genomes.org). To generate the genotype of an individual, we generate two haplotypes according to the haplotype frequencies.

We test K = 4 related traits with a compound-symmetry correlation matrix and consider two covariates: a standard normal covariate *z*_1_ and a binary covariate *z*_2_ with *P*(*z*_2_ = 1) = 0.5. We generate trait values based on genotypes by using the following models:

$$\begin{array}{cc} y_{k} & =0.5z_{1}+0.5z_{2}+\eta_{k}+\epsilon_{k}\;\;\;k=1,2,3,4 \end{array}$$

where ***ϵ*** = (*ϵ*_1_, *ϵ*_2_, *ϵ*_3_, *ϵ*_4_) is zero-mean normal with variances 1 and correlation *ρ*. We set the magnitude of correlation |*ρ*| to 0.2, 0.5, and 0.8, and the signs of symmetric location of covariate matrix are randomly chosen from (-1,1). ***η*** = (*η*_1_, *η*_2_, *η*_3_, *η*_4_) are contributions from a set of genotypic variants, which are simulated as follows.

For type I error, phenotypes are generated under the null model i.e. ***η*** = 0. To evaluate power, we randomly choose one common variant and *n*_*c*_ (20%) rare variants as casual variants. We assume that all the *n*_*c*_ rare causal variants have the same heritability and the heritability of the common causal variant is twice of the heritability of rare causal variants. That is, we model the genotypic variants’ contribution to disease risk as $\eta_{k}=\beta_{c}x_{c}+\sum_{j=1}^{n_{c}}\beta_{kj}x_{j},k=1,\cdots ,4$ where *x*_*c*_ and *x*_*j*_ denote the common variant and rare variant, respectively. *β*_*c*_ and *β*_*kj*_ represent the corresponding effect size. Let *h* and *h*_*k*_ denote the heritability of all the causal variants for all the *K* traits and for the *k*^*th*^ trait, respectively. We generate *K* random numbers *t*_1_, ⋯, *t*_*K*_ from a uniform distribution between 0 and 1, and the heritability of *k*^*th*^ trait denotes $h_{k}=ht_{k}/\sum_{k=1}^{K}t_{k}$. For the *k*^*th*^ trait, we assign the effect size of common variants

$$\beta_{c}=\sqrt{\frac{h_{k}}{\text{var}\left(x_{c}\right)\left(1-h_{k}\right)\left(1+R\right)}}$$ (7)

and the magnitude of the effect of rare variants

$$|\beta_{kj}|=\sqrt{\frac{h_{k}R}{\text{var}\left(x_{c}\right)\left(1-h_{k}\right)n_{c}\left(1+R\right)}}$$ (8)

where *R* denotes the ratio of the heritability of rare causal variants to the heritability of the common causal variant.

For power comparisons, we conducted simulations under the four scenarios: each time only the first *L* traits are associated with the set of variants, *L* = 1, 2, 3, 4, respectively. Intuitively, in the first scenario (L = 1), when only the first trait is associated with the variants set, the minP method (it equals to test the first trait alone) may have good performance. However, we will show that by simultaneously testing correlated null traits, our proposed method (TOW-CM) could actually improve the detection power compared to test the first trait alone. When there are multiple correlated traits that are associated with the rare variants set, the proposed TOW-CM would offer vastly improved detection power than the minimum p-value based approach. In each scenario, we also consider different percentage of risk variants for rare variants.

## Simulation results

Table 1 summarizes the estimated type I error rates of our method TOW-CM with other three comparable methods under different significance levels and different magnitude of trait correlation |*ρ*|. The type I error rates are evaluated using 10000 replicated samples and the P-values are estimated using 10000 permutations for TOW-CM and minP. For the 10000 replicated samples, the 95% confidence intervals (CIs) for the estimated type I error rates of nominal levels 0.05, 0.01, and 0.001 are (0.046, 0.054), (0.008, 0.012), and (0.0004, 0.0016), respectively. From this table, we can see that all of the estimated type I error rates are either within 95% CIs or close to the bound of the corresponding 95% CIs, which indicate that the type I error rates of all methods are controlled under all considered scenarios.

**Table 1:** The estimated type I error rates for TOW-CM, minP, MANOVA and MSKAT.

	*α* = 0.05				
Sample Size		TOW-CM	minP	MANOVA	MSKAT
1000	|*ρ*| = 0.2	0.054	0.055	0.055	0.045
	|*ρ*| = 0.5	0.054	0.052	0.054	0.046
	|*ρ*| = 0.8	0.052	0.049	0.053	0.048
2000	|*ρ*| = 0.2	0.050	0.053	0.052	0.049
	|*ρ*| = 0.5	0.048	0.050	0.052	0.049
	|*ρ*| = 0.8	0.048	0.053	0.052	0.051
3000	|*ρ*| = 0.2	0.049	0.051	0.052	0.050
	|*ρ*| = 0.5	0.053	0.055	0.050	0.049
	|*ρ*| = 0.8	0.048	0.049	0.053	0.050
	*α* = 0.01				
1000	|*ρ*| = 0.2	0.012	0.010	0.010	0.009
	|*ρ*| = 0.5	0.011	0.008	0.011	0.010
	|*ρ*| = 0.8	0.012	0.010	0.010	0.007
2000	|*ρ*| = 0.2	0.012	0.012	0.011	0.008
	|*ρ*| = 0.5	0.010	0.012	0.010	0.009
	|*ρ*| = 0.8	0.010	0.010	0.011	0.010
3000	|*ρ*| = 0.2	0.010	0.013	0.010	0.011
	|*ρ*| = 0.5	0.012	0.011	0.010	0.010
	|*ρ*| = 0.8	0.010	0.011	0.011	0.010
	*α* = 0.001				
1000	|*ρ*| = 0.2	0.0014	0.0010	0.0012	0.0008
	|*ρ*| = 0.5	0.0004	0.0008	0.0010	0.0007
	|*ρ*| = 0.8	0.0010	0.0011	0.0010	0.0009
2000	|*ρ*| = 0.2	0.0013	0.0012	0.0007	0.0012
	|*ρ*| = 0.5	0.0016	0.0012	0.0007	0.0008
	|*ρ*| = 0.8	0.0011	0.0010	0.0010	0.0005
3000	|*ρ*| = 0.2	0.0005	0.0013	0.0008	0.0009
	|*ρ*| = 0.5	0.0011	0.0012	0.0008	0.0011
	|*ρ*| = 0.8	0.0011	0.0011	0.0009	0.0005

**Fig 1:**
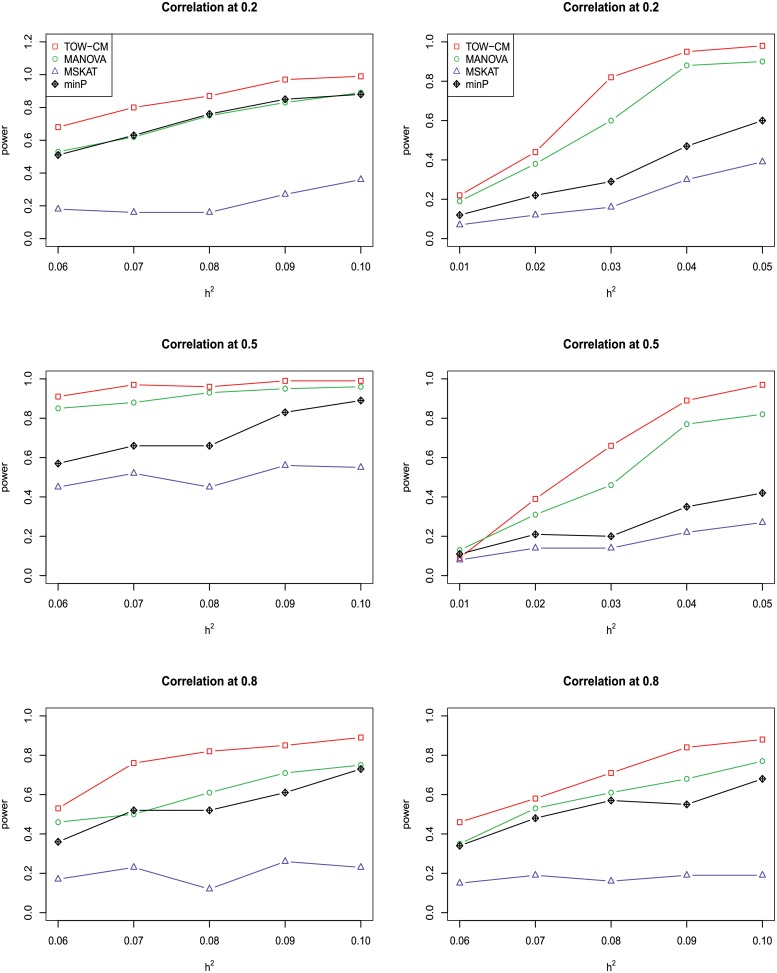
Power comparison of four tests as a function of heritability for four continuous traits with the magnitude of correlation at 0.2, 0.5 and 0.8, respectively. All four traits are associated with the gene for the left panel and only the first three traits are associated with the gene for the right panel. Sample size is 1,000 and 20% of rare variants are causal. All causal variants are risk variants. The powers are evaluated at a significance level of 0.05.

**Fig 2:**
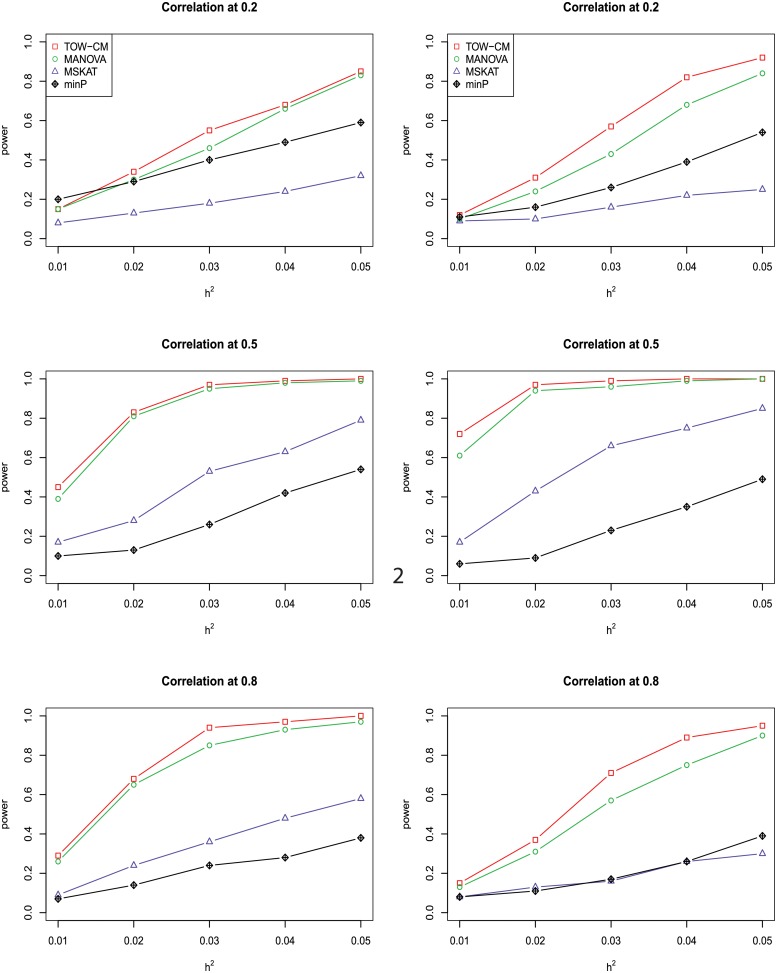
Power comparison of four tests as a function of heritability for four continuous traits with the magnitude of correlation at 0.2, 0.5 and 0.8, respectively. All four traits are associated with the gene for the left panel and only the first three traits are associated with the gene for the right panel. Sample size is 1,000 and 20% of rare variants are causal variants among which 90% of causal variants are risk variants and 10% of causal variants are protective variants. The powers are evaluated at a significance level of 0.05.

**Fig 3:**
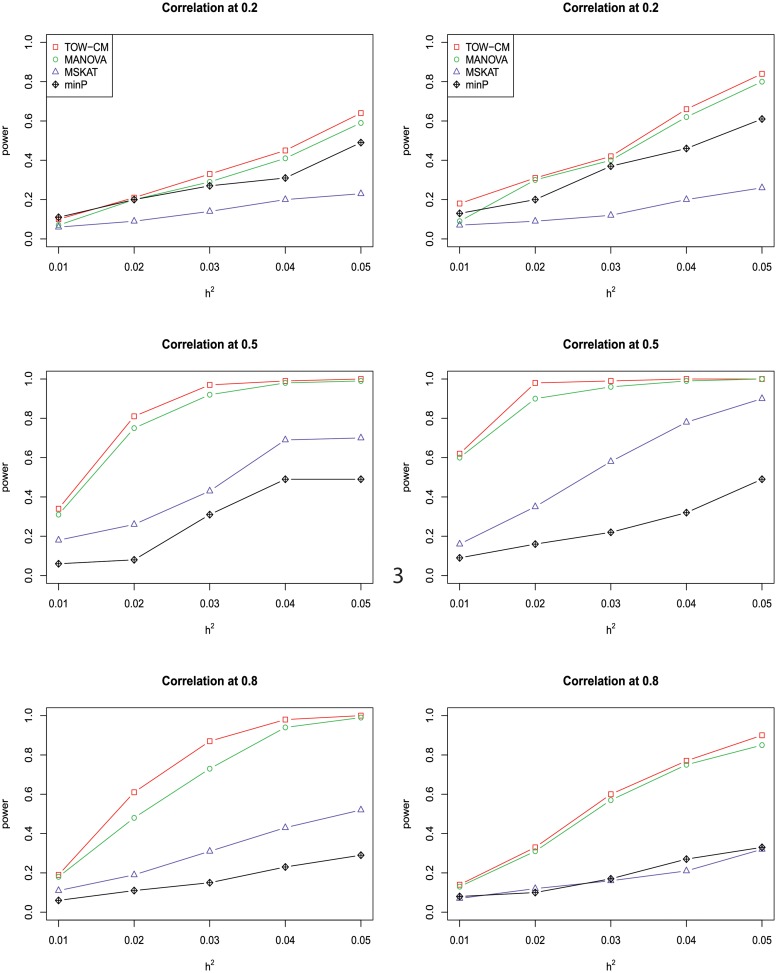
Power comparison of four tests as a function of heritability for four continuous traits with the magnitude of correlation at 0.2, 0.5 and 0.8, respectively. All four traits are associated with the gene for the left panel and only the first three traits are associated with the gene for the right panel. Sample size is 1,000 and 20% of rare variants are causal among which 80% of causal variants are risk variants and 20% of causal variants are protective variants. The powers are evaluated at a significance level of 0.05.

**Fig 4:**
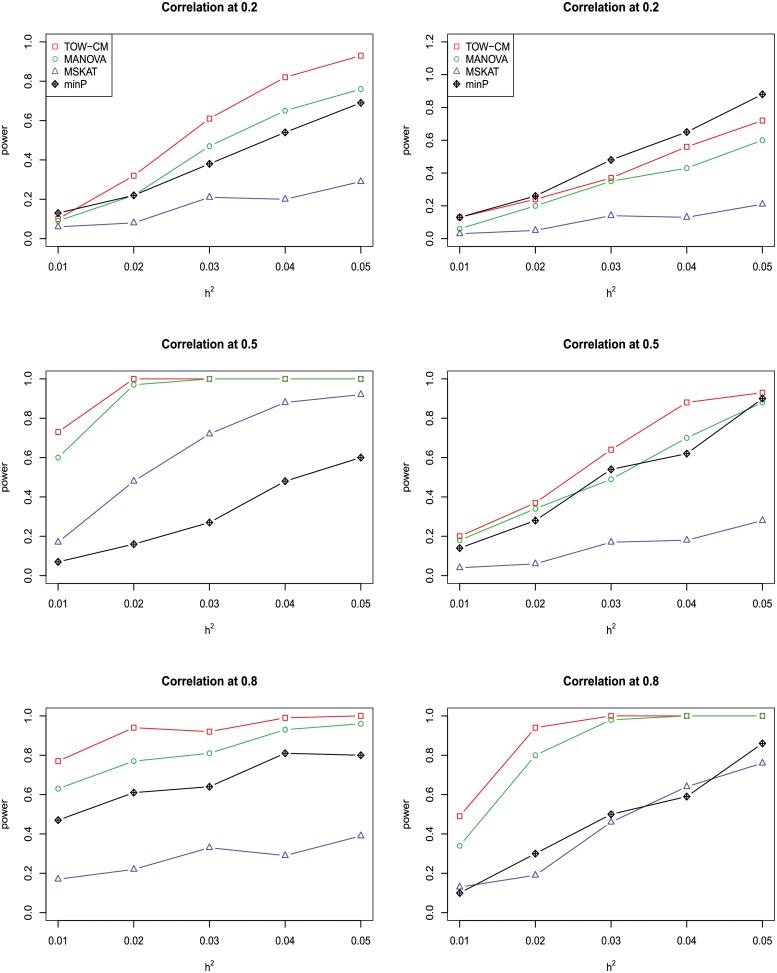
Power comparison of four tests as a function of heritability for four continuous traits with the magnitude of correlation at 0.2, 0.5 and 0.8, respectively. Only the first two traits are associated with the gene for left panel and only the first traits are associated with the gene for right panel. Sample size is 1,000 and 20% of rare variants are causal variants. All causal are risk variants. The powers are evaluated at a significance level of 0.05.

**Fig 5:**
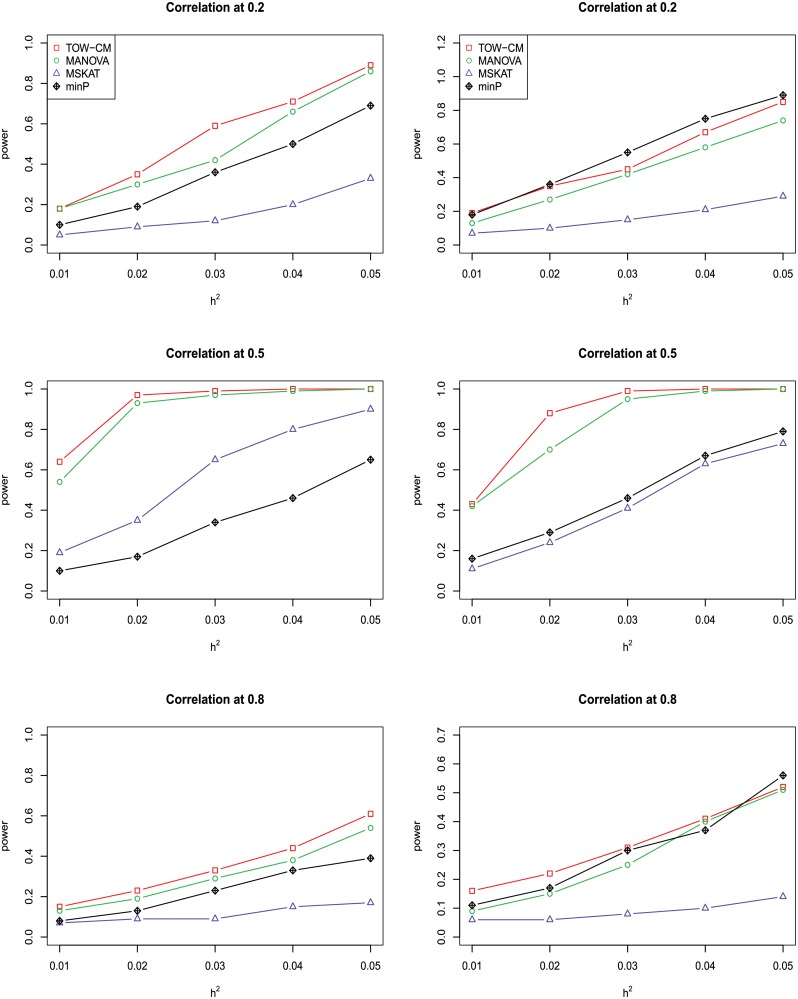
Power comparison of four tests as a function of heritability for four continuous traits with the magnitude of correlation at 0.2, 0.5 and 0.8, respectively. Only the first two traits are associated with the gene for left panel and only the first traits are associated with the gene for right panel. Sample size is 1,000 and 20% of rare variants are causal. 90% of causal are risk variants and 10% of causal are protective variants. The powers are evaluated at a significance level of 0.05.

**Fig 6:**
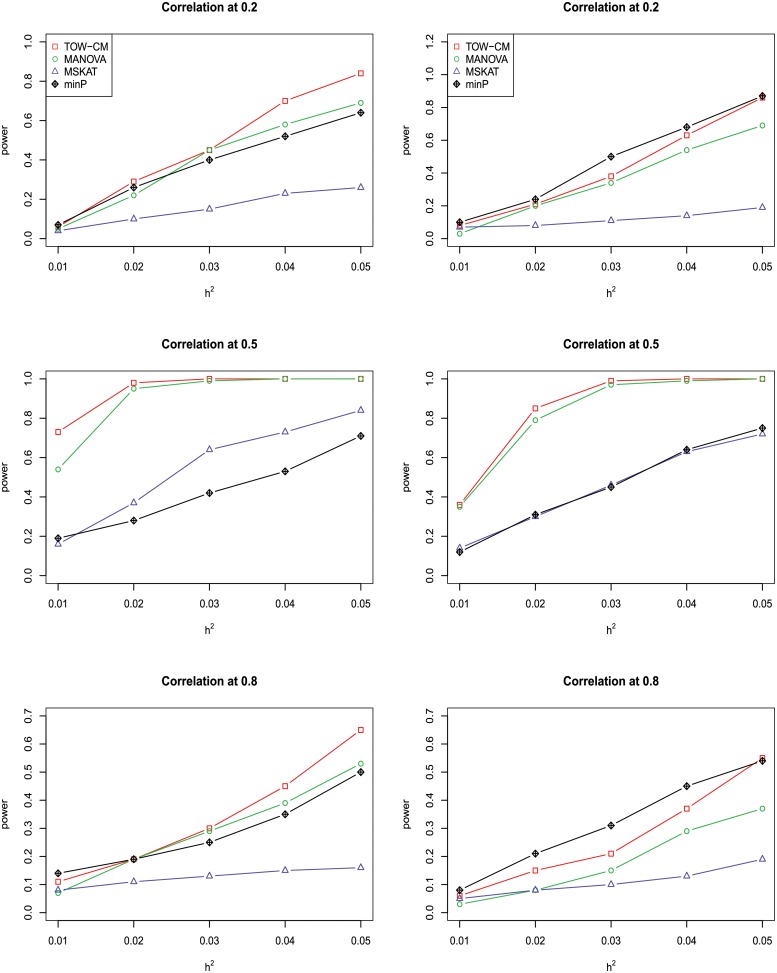
Power comparison of four tests as a function of heritability for four continuous traits with the magnitude of correlation at 0.2, 0.5 and 0.8, respectively. Only the first two traits are associated with the gene for left panel and only the first traits are associated with the gene for right panel. Sample size is 1,000 and 20% of rare variants are causal among which 80% of causal variants are risk variants and 20% of causal variants are protective variants. The powers are evaluated at a significance level of 0.05.

In power comparisons, the P-values of TOW-CM, minP are calculated using 1000 permutations, while the P-values of MANOVA and MSKAT are calculated by asymptotic distributions. The powers of all the four tests are evaluated using 1000 replicated samples at a nominal significance level of 0.05. Figs 1–6 present the power under significance level 0.05 for *L* = 4, 3, 2, 1 respectively.

These figures show the power comparisons of the four tests (TOW-CM, MANOVA, MSKAT and minP). Power is a function of the total heritability based on three cases (all causal are risk variants, 90% causal are risk variants, and 80% causal are risk variants) for each specific scenario L. These figures show that TOW-CM is consistently the most powerful test among the four tests, and MANOVA is the second most powerful test when genotypes of genetic variants have impact on more than 1 traits. MSKAT is consistently less powerful than the other two multivariate tests (TOW-CM and MANOVA) most likely because there are only 8% variants with MAF in the range of (0.01,0.035) in Sgene which the simulations are based on. Similar to SKAT, MSKAT will lose power when the MAF of causal variants are not in the range (0.01,0.035) [23]. The minP method is consistently less powerful than TOW-CM and MANOVA because they ignore the traits’ dependence by directly using minimum of the P-values of testing the four single traits. Overall, we can see that they suffer power loss when the correlations among traits increase.

An interesting scenario is one in which only the first trait is associated with the variants set and all the others are null traits (L = 1). Stephens [24] and Wu et al. [25] have reported that joint testing by incorporating a correlated null trait could improve the power for testing association of a common variant. When only the first trait is associated with the variants set, minP is either the most powerful test or has similar power to the most powerful test especially in the case of both causal variants under weak traits correlation (|*ρ*| = 0.2). The TOW-CM and MANOVA statistic could benefit from increased traits correlations, and offer vastly improved power by incorporating strongly correlated null traits. Thus, our results verify the conclusion of [24] and [25].

Overall, we can see that the proposed TOW-CM is an attractive approach that provides good power in most of the scenarios.

## Application to the COPDGene

Chronic obstructive pulmonary disease (COPD) is one of the most common lung diseases characterized by long term poor airflow and is a major public health problem [26]. The COPDGene Study is a multi-center genetic and epidemiologic investigation dedicated to studying COPD [27]. Participants in the COPDGene Study gave consent for the use of data collected during the study in downstream analyses. This study is sufficiently large and appropriately designed for analysis of COPD. In this study, we consider more than 5000 non-Hispanic Whites (NHW) participants where the participants have completed a detailed protocol, including questionnaires, pre- and post-bronchodilator spirometry, high-resolution CT scanning of the chest, exercise capacity (assessed by six-minute walk distance), and blood samples for genotyping. The participants were genotyped using the Illumina OmniExpress platform. The genotype data have gone through standard quality-control procedures for genome-wide association analysis detailed at http://www.copdgene.org/sites/default/files/GWAS_QC_Methodology_20121115.pdf.

Based on the literature studies of COPD [28, 29], we selected 7 key quantitative COPD-related phenotypes, including FEV1 (% predicted FEV1), Emphysema (Emph), Emphysema Distribution (EmphDist), Gas Trapping (GasTrap), Airway Wall Area (Pi10), Exacerbation frequency (ExacerFreq), Six-minute walk distance (6MWD), and 4 covariates, including BMI, Age, Pack-Years (PackYear) and Sex. EmphDist is the ratio of emphysema at -950 HU in the upper 1/3 of lung fields compared to the lower 1/3 of lung fields where we did a log transformation on EmphDist in the following analysis, referred to [28]. In the analysis, participants with missing data in any of these phenotypes were excluded.

To evaluate the performance of our proposed method on a real data set, we applied all of the 4 methods (TOW-CM, MANOVA, MSKAT and minP) to the COPD associated genes or genes containing significant single-nucleotide polymorphisms (SNPs) in NHW population with COPD-related phenotypes [30]. In the analysis, we first removed the missing data in any genotypic variants and then adjusted each of the 7 phenotypes for the 4 covariates using linear models. In the analysis, participants with missing data in any of the 11 variables were excluded. Therefore, a complete set of 5,430 individuals across 50 genes were used in the following analyses. In order to compare these methods, we adopted the commonly used 10^7^ permutations for TOW-CM and minP methods. For this verification study, we use 0.05 as the significance level for MANOVA, MSKAT and TOW-CM methods and use Bonferroni corrected significance level 0.05/7 = 7.14 × 10^−3^ for minP methods since this method perform association tests across each trait, respectively. The results are summarized in Table 2. From Table 2, we can see that TOW-CM identified 14 genes, minP identified 14 genes, MANOVA identified 12 genes and MSKAT identified 4 genes. Among these four methods, TOW-CM identified the most significant genes where all of these 14 genes had previously been reported to be in association with COPD by eligible studies [7, 30], among which 5 genes (LOC105377462,CHRNA3, CHRNA5,HYKK,IREB2) are statistically significant if we use a more stringent cut-off 1.00 × 10^−3^ for a multiple testing issue with 50 genes in total. Because the MAFs of most variants are not in the range of (0.01,0.035) which is a range favoring MSKAT, MSKAT performs worse than the other three comparable methods (Yang et al. 2017). TOW-CM and minP perform better than MANOVA, which is perhaps because only a proportion of phenotypes are associated with COPD. The method minP missed some genes in comparision to our method TOW-CM, it may because the method minP ignores the correlation between these seven phenotypes.

**Table 2:** The p-values of significant genes in the genetic association analysis for COPD using these four different methods.

Chr	Genes	Range of MAF	minP (0.05 / 7)	MANOVA (0.05)	MSKAT (0.05)	TOW-CM (0.05)
1	EPHX1	(0.0214, 0.4620)	0.0197	0.6055	0.5890	0.6257
1	IL6R	(0.1680, 0.4398)	0.2646	0.5214	0.5163	0.5148
1	MFAP2	(0.1789, 0.4842)	0.0753	0.6986	0.9926	0.6869
1	TGFB2	(0.0139, 0.4858)	**7.23** × **10**^**−4**^	0.2282	0.1831	**3.47** × **10**^**−2**^
2	HDAC4	(0.0147, 0.4906)	0.0468	0.3393	0.2197	0.5026
2	SERPINE2	(0.0143, 0.4642)	0.4671	0.9797	0.7706	0.9010
2	SFTPB	(0.0784, 0.4766)	0.0738	0.1017	0.1669	0.3921
2	TNS1	(0.0128, 0.4936)	0.00727	**4.63 × 10**^**− 2**^	0.2095	**2.65 × 10**^**− 2**^
3	MECOM	(0.0099, 0.4957)	0.0359	0.9878	0.7211	0.9735
3	RARB	(0.0278, 0.4942)	0.0491	0.1988	0.7469	0.3973
4	LOC105377462	(0.0190, 0.4933)	**0.00**	**6.28 × 10**^**− 3**^	0.8310	**0.00**
4	FAM13A	(0.0279, 0.4968)	**1.08 × 10**^**− 5**^	0.2169	0.0939	0.3925
4	GC	(0.0511, 0.4397)	0.1743	0.1875	0.6499	0.5257
4	GSTCD	(0.0343, 0.3872)	**3.6 × 10**^**− 6**^	**5.63 × 10**^**− 5**^	0.1376	**3.30 × 10**^**− 2**^
4	HHIP	(0.0368, 0.4984)	0.0150	**3.64 × 10**^**− 2**^	0.4131	**1.95 × 10**^**− 3**^
5	HTR4	(0.0396, 0.4889)	0.0487	0.6622	0.6512	0.8906
5	SPATA9	(0.1059, 0.4077)	0.1145	0.3118	0.5198	0.1964
6	TNF	(0.0259, 0.0809)	0.0320	0.1627	0.1542	0.3077
6	ZKSCAN3	(0.0137, 0.3036)	0.4990	0.8575	0.9083	0.8793
6	AGER	(0.0442, 0.1830)	**3.25 × 10**^**− 4**^	**2.27 × 10**^**− 3**^	**9.31 × 10**^**− 4**^	**9.57 × 10**^**− 3**^
6	ARMC2	(0.0187, 0.4695)	0.1618	0.2481	0.1474	0.6233
6	NCR3	(0.0133, 0.0899)	0.0735	0.4641	0.4145	0.5892
6	SOX5	(0.0193, 0.4972)	0.0764	0.8376	0.6845	0.6386
10	LRMDA	(0.0094, 0.4956)	0.0394	0.4102	0.7190	0.3260
10	CDC123	(0.0240, 0.4561)	0.0138	0.6846	0.4097	0.8836
10	GSTO2	(0.0538, 0.4547)	**4.0 × 10**^**− 7**^	**1.36 × 10**^**− 6**^	0.1387	0.8731
10	SFTPD	(0.0186, 0.4367)	0.3699	0.9997	0.9767	0.9751
11	GSTP1	(0.3351, 0.3452)	**6.60 × 10**^**− 3**^	0.7053	0.1211	0.5043
11	MMP1	(0.0519, 0.3916)	0.1665	0.8614	0.6557	0.9449
11	MMP12	(0.0541, 0.1439)	0.4073	0.9512	0.7372	0.8941
12	LRP1	(0.0271, 0.4071)	0.0144	0.4530	0.5326	0.1812
12	BICD1	(0.0224, 0.4984)	0.3045	0.3856	0.2186	0.4076
12	CCDC38	(0.0783, 0.4669)	0.3525	0.1151	0.5888	0.2316
14	SERPINA1	(0.0212, 0.4171)	0.0254	0.6161	0.0816	0.3506
14	SERPINA3	(0.1076, 0.4907)	0.4336	0.8567	0.6572	0.7375
15	CHRNA3	(0.0515, 0.4234)	**4.0 × 10**^**− 7**^	**1.36 × 10**^**− 6**^	0.1387	**0.00**
15	CHRNA5	(0.2170, 0.4178)	**1.6 × 10**^**− 6**^	**3.27 × 10**^**− 7**^	**5.77 × 10**^**− 6**^	**0.00**
15	HYKK	(0.1070, 0.4139)	**0.00**	**1.42 × 10**^**− 7**^	**0.0152**	**0.00**
15	IREB2	(0.1577, 0.4287)	**3.6 × 10**^**− 6**^	**5.63 × 10**^**− 5**^	0.1376	**1.10 × 10**^**− 4**^
15	THSD4	(0.0115, 0.4944)	0.00725	0.0798	0.8496	0.0669
16	CFDP1	(0.0424, 0.4139)	0.0991	0.9474	0.7127	0.9772
17	TIMP2	(0.0403, 0.4950)	**1.03 × 10**^**− 3**^	0.1828	0.3702	0.6836
19	CYP2A6	(0.2386, 0.2505)	**3.10 × 10**^**− 3**^	**1.15 × 10**^**− 2**^	**3.47 × 10**^**− 2**^	**2.85 × 10**^**− 2**^
19	EGLN2	(0.0465, 0.3712)	0.00870	0.3913	0.3705	**4.36 × 10**^**− 2**^
19	MIA	(0.0459, 0.0691)	0.1152	0.0647	0.4979	**3.78 × 10**^**− 2**^
19	RAB4B	(0.1374, 0.4273)	**4.00 × 10**^**− 4**^	**4.60 × 10**^**− 2**^	0.7020	**2.42 × 10**^**− 3**^
19	TGFB1	(0.0274, 0.4899)	0.0418	0.3039	0.7122	0.4531
20	MMP9	(0.0412, 0.4234)	0.0896	0.8143	0.79926	0.7949
21	KCNE2	(0.1172, 0.2778)	0.1283	0.3687	0.3776	0.6938
22	HMOX1	(0.0530, 0.4270)	0.0109	0.1936	0.1181	0.1107

Note: significance level 0.05 for MANOVA, MSKAT, TOW-CM, and 0.05/7 for minP.

## Discussion

GWAS have identified many variants with each variant affecting multiple phenotypes, which suggests that pleiotropic effects on human complex phenotypes may be widespread. Also, recent studies have shown that complex diseases are caused by both common and rare variants [14, 16, 19]. Therefore, statistical methods that can jointly analyze multiple phenotypes for common or/and rare variants have advantages over analyzing each phenotype individually or only considering for common variants (GWAS). In this article, we propose TOW-CM method to perform multivariate analysis for multiple phenotypes in association studies based on the following reasons: (1) complex diseases are usually measured by multiple correlated phenotypes in genetic association studies; (2) there is increasing evidence showing that studying multiple correlated phenotypes jointly may increase power for detecting disease associated genetic variants, and (3) there is a shortage of gene-based approaches for multiple traits. Simulation results show that TOW-CM has correct type I error rates and is consistently more powerful in comparision to the other three tests. The real data analysis results show that TOW-CM has excellent performance in identifying genes associated with complex disease with multiple correlated phenotypes such as COPD.

One disadvantage of TOW-CM is that the test statistic does not have an asymptotic distribution and a permutation procedure is needed to estimate its P-value, which is time consuming compared to the methods whose test statistics have asymptotic distributions. To save time when applying TOW-CM to genetic association studies, we can use the “step-up” procedure [31] to determine the number of permutations, which can show evidence of association based on a small number of permutations first (e.g. 1,000) and then a large number of permutations are used to test the selected potentially significant genes. Specifically, for the analysis of real data, the computation time of p-value estimation of TOW-CM for all genes was about three days using our R program on 50 Dell PowerEdge C6320 servers. Each server has two 2.4GHz Intel Xeon E5-2680 v4 fourteen-core processors and 600 MB average memory. We also uploaded the R program on GitHub, https://github.com/Jianjun-CN/TOW-CM/blob/master/R%20Code Furthermore, TOW-CM method can not only be used for gene-based association studies, but also can be extended to transcriptome-wide association study (TWAS), which needs further investigations.
